# Acculturation, Dietary Acceptability, and Diabetes Management among Chinese in North America

**DOI:** 10.3389/fendo.2013.00108

**Published:** 2013-08-27

**Authors:** Feiyue Deng, Anran Zhang, Catherine B. Chan

**Affiliations:** ^1^Department of Agricultural, Food and Nutritional Science, University of Alberta, Edmonton, AB, Canada; ^2^Department of Physiology, University of Alberta, Edmonton, AB, Canada

**Keywords:** dietary acculturation, immigrants, type 2 diabetes, ethnic Chinese, nutritional interventions

## Abstract

Immigrants to a new country face many challenges when diagnosed with type 2 diabetes, a chronic disease with a complex treatment involving both medical and behavioral interventions. These challenges will depend upon the extent to which the patient has adapted to the new country’s social and cultural norms, as well as individual factors such as age, education, and gender. This adaptation is termed acculturation. With respect to nutritional interventions for type 2 diabetes, uptake and adherence over the long term will depend upon overall health literacy, the cultural acceptability of the recommended diet. This review has focused on acculturation and its effects on diabetes management in ethnic Chinese in North America as an example of one populous minority and the challenges faced in adopting nutritional recommendations. Research directions and practical considerations are suggested.

## Introduction

Changes in health status following immigration have been noted, with health advantages disappearing over time (1) and increasing prevalence of obesity and associated metabolic diseases such as type 2 diabetes (T2D) (2, 3); this change over time is known as the “healthy immigrant effect.” This suggests that adaptation to the new environment and culture is a risk factor for diabetes. Immigrants may also face obstacles to optimized treatment of complex chronic diseases due to many factors; lack of optimization of interventions based on culture may be a major barrier.

As will be described further, the adaptation occurring following immigration is termed *acculturation*, which has been defined as “the process by which an ethnic group, usually a minority, adopts the cultural patterns including beliefs, religion, and language of a dominant group” (4). At the group level, the acculturating group (or society as a whole) adopts physical, economic, cultural, and political changes while at the individual level, change is manifested in attitudes, beliefs, values, and behaviors (for example dietary behaviors and eating patterns) (4). It is proposed that acculturation is affected by personal factors such as age, gender, and socio-economic status, the duration of stay in the host country and the traditions of the original culture (4). As reviewed by Sanou et al. (1), significant knowledge gaps exist regarding the interrelationship between immigration, acculturation, and health status in Canada but acculturation may affect an individual’s ability to fully embrace treatment (1), which for T2D includes a complex regime of self-care activities such as managing diet, increasing physical activity, monitoring blood glucose, taking medication, and being vigilant for co-morbidities (5). Because diet is closely tied to culture, adhering to a prescribed nutrition regimen may be even more difficult than for the dominant culture.

A major immigrant group in Canada is ethnic Chinese. According to the 2011 census, first generation immigrants make up about 20% of Canada’s population. Ethnic Chinese are the second largest population of first generation Canadians and are also the second largest visible minority in Canada overall, comprising 4.0% of the total population (6). Only five published papers regarding health of Chinese immigrants to Canada were found in the literature (1). However, several studies have examined the association between the prevalence of chronic diseases and acculturation among Asian immigrants to the United States. American-Chinese have a higher prevalence of diabetes compared with both native Americans and their peers in China (7).

The purpose of this review is first to describe general barriers to dietary adherence in people with T2D. We will then summarize what is known about the influence of several acculturation indicators (diet, language, length of stay in North America) on T2D management behaviors in Chinese immigrants to North America and the effects of acculturation on diabetic management in different genders and family context. Avenues for improving diabetes nutrition therapy for ethnic Chinese, as a model for immigrant populations to Canada, will be suggested.

## Adherence to Nutrition Interventions in Type 2 Diabetes

Nutrition therapy is a critical component of care for people with T2D and has the potential to lower glycated hemoglobin (A1c, a marker of blood glucose control) by 1–2% (5). However, dietary management is perceived to be the most difficult to achieve of all the aspects of self-care (8, 9). Studies conducted in various countries around the world show that adherence to dietary recommendations is poor among diabetes patients (10–12). Data from the US-based National Health and Examination survey (NHANES) indicate that protein, saturated fat, and fiber recommendations were met by 65, 28, and 18% of those with diabetes, respectively (13). Thus, the accumulated evidence suggests that diabetes patients have difficulty implementing recommended dietary changes into their daily routines and lifestyle. Family physicians and other health care providers may be enthusiastic supporters of lifestyle modifications but are lacking practical resources to help their patients with T2D achieve these recommendations (14). Clinical Practice Guidelines produced by national and international organizations (5, 15, 16) provide specific recommendations but do not address how guidelines can be met except in a general way.

A major part of the uncertainty regarding adherence to dietary treatment and its “success” or “failure” is related to our lack of understanding of the extent to which patients adopt and are able to maintain changes that they make in their dietary intake. For example, a systematic review (17) of dietary advice for the treatment of T2D found only 36 papers reporting on 18 studies that met the inclusion criteria for this review. The authors noted that many studies failed to measure and report adherence to the intervention, limiting their ability to make conclusions as to the relative efficacy of interventions. Failure to adhere to nutrition advice may actually reflect a range of factors ranging from social and ecological to personal and familial factors that limit patients’ ability to implement changes in diet. Even less is known about how minority or immigrant populations respond to interventions for chronic diseases compared with the general population. Osei-Assibey et al. (18) conducted a systematic review and found only 19 publications comparing minority populations within randomized control trials aimed at weight loss in the obese. All of the studies were conducted in the United States and focused mainly on African-Americans. In the Diabetes Prevention Program, an ethnically representative sample was studied and ethnic × gender differences in response to the intervention were noted, with female African-Americans losing less weight than their white counterparts (19). However, the authors of that study noted that weight loss was still greater than in most other published reports and attributed the effect to the intervention design, which included tailoring foods and cooking methods to ethnic groups, among other strategies (19).

## Focus on Cultural and Social Acceptability of Prescribed Diets

The need to recognize cultural diversity in diabetes treatment is recognized by the International Diabetes Federation (16) and countries with large immigrant populations and multi-cultural societies (5). When optimizing dietary patterns in order to treat disease, the food culture of the society and the individual should be considered in order to maximize the acceptability of the treatment. Choosing unfamiliar foods from a different ethnic heritage might make dietary adherence to diabetes guidelines more complicated and could contribute to low adherence rates, while acceptability of a recommended diet could increase adherence. Therefore, understanding the personal and cultural barriers that are associated with dietary adherence faced by people with diabetes could contribute to a future intervention program.

Food acceptability is widely referred to terms such as palatability, liking/disliking, food preferences, and pleasantness/unpleasantness (20). For example, the Dietary Approaches to Stop Hypertension (DASH) diet has been evaluated for the acceptability of reduced salt in foods (21). However, food acceptability also has social and cultural connotations. Holm’s group (22) divides social and cultural acceptability into four dimensions including liking of the diet, social eating events, practical matters of implementation (shopping, cooking, eating), and relationship of the diet to the desired outcome (e.g., weight loss). Similarly in diabetes populations, it is predicted that a person will be better able to follow the prescribed diet when the foods specific for diabetes are available at an affordable price with easy access and are acceptable culturally, socially, and personally. The association between these factors and dietary adherence is not widely studied in diabetes. Understanding these factors would help to plan intervention programs that would be more effective and convenient for diabetes patients. CDA’s Clinical Practice Guidelines (5) do advise that the health care team consider cultural and personal preference when formulating diet plans for clients but how well these preferences are incorporated is not widely studied.

Few studies assess the effect of cultural acceptability on dietary adherence. Interestingly, prescribing a diet outside of the cultural norm was persistently associated with participants’ feeling that the diet was difficult to incorporate into the family’s eating patterns or with social interactions. There were also perceptions of increased investment of time and money for food preparation and difficulty in incorporating into traditional dishes (23). Results suggest that diabetic individuals have difficulty altering their foods habits and often tend to consume traditional foods that are high in fat and sugar (24, 25). Data from our group suggest that higher cultural and social acceptability of an individual’s prescribed diabetes diet correlates with improved blood glucose control (26); however, these results were obtained from a cross-sectional study of predominantly white participants. A study of ethnic minority or immigrant participants would help to clarify the relative acceptability of diabetes diets and the relationship to health outcomes. Another study examining diabetes dietary satisfaction also found improved A1c when participants expressed better ability to implement a diabetes diet, including with regard to social situations (27), but cultural considerations were not addressed. Diabetes educators (28) and people with diabetes (24, 29) report that not wanting to give up ethnic foods is a barrier to adherence whereas when dietary recommendations are flexible and adapted to a specific culture, better control of blood glucose may be achieved (30). Modifying traditional foods to make them healthier may also be of benefit (31). It has been suggested that new immigrants should retain their traditional healthy eating patterns while adopting the healthy components of the dietary practices of their host country (32). Health professionals could decide whether to focus more on the former or the latter, based on specific dietary acculturation of an individual or community. For example, if an immigrant has a very low acculturation level, and his diet remains traditional, more attention should be paid to retaining his traditional healthy dietary practices instead of letting him incorporate the healthy eating patterns of the host country, which may result in low acceptability and low adherence.

## Acculturation, Dietary Acculturation, and Diabetes Management

For an immigrant in North America, the degree of acculturation influences many aspects of life, such as access to health services organizations and physical activity facilities as well as healthy eating behaviors. Therefore, the degree to which individuals are acculturated into the host culture is predicted to influence their ability to adapt to the requirements of a therapeutic nutritional pattern upon diagnosis of a chronic disease like T2D. Acculturation can be quantified using instruments such as the Suinn-Lew Asian Self-Identity Acculturation (SL-ASIA) scale, which examines responses to statements in several dimensions including ethnic identity, social interactions, language and cultural preference, country of birth and pride for one’s culture (7). The term “dietary acculturation” specifically refers to the process of adopting the eating patterns and food choices of their new environment (33). Dietary acculturation is multidimensional, dynamic, and complex, and does not appear to be a linear process. Instead of moving predictably from traditional to acculturated, immigrants may “retain and find new ways to use traditional foods, exclude others, and/or consume new foods” (32).

When Chinese people immigrate to North America, dietary patterns change as a result of acculturation and availability of specific foods. More than half of respondents changed their diet after immigration from China to the USA, with increased consumption of western and reduced consumption of traditional Chinese foods from all food groups, as well as increased consumption of sweets and soft drinks (34). Lack of availability of traditional Chinese food at competitive prices with western foods is an important factor (35). Of the three daily meals, breakfast is usually the first to be acculturated, with inclusion of foods such as oatmeal, milk, bagels, and cream cheese whereas noodles or rice remain staples of the lunch and supper meals (36). This dietary acculturation pattern is likely a risk factor for obesity and T2D. As acculturation progresses, intake of processed, ready-to-eat western foods increases because of lack of familiarity with western cooking methods (36).

In general, dietary acculturation has been shown to have negative influence on the health status of immigrants, placing them at elevated risk for chronic diseases related to diet (32). Using Canadian data from several nationally representative surveys, there was a similar (37) diabetes prevalence between the Chinese-Canadian population and the general Canadian population. However, a prospective study with median 6 years of follow-up of diabetes incidence in Ontario, Canada showed that Chinese had 87% greater risk than white subjects (38). The ethnic Chinese were also younger at diagnosis and developed diabetes at a lower BMI than Caucasian Canadians (38). Asian Americans have also been reported to have increased risk of diabetes (39) although a recent US study indicated similar risk to whites for incident diabetes over 1 year of follow-up (40). Thus, ethnic Chinese in North America are a sizable population with diabetes prevalence equal to or greater than the general population. From both economic and personal perspectives, there is a strong rationale for optimizing diabetes treatment, particularly related to nutrition, in this group.

Nutrition therapy is an important part of diabetes treatment (5) but it is a challenging task to change and maintain one’s diet. Immigrant groups usually have different dietary patterns than native Canadians, so it is likely to be even harder for them to adhere to a typical Canadian food guide or menu plan. Application of dietary acculturation to specific immigrant groups holds special challenges and self-care during chronic disease is better facilitated when cultural competence and congruency is incorporated in the management approach (41). In Canada, the importance of providing individualized behavioral treatment taking cultural factors into account has been noted by researchers (42) as well as in the CDA Clinical Practice Guidelines (5). Dietary acculturation occurs to varying extents in people, with different combinations of traditional and western foods included in the diet, and appears to occur more in younger immigrants (34, 43); therefore, for older adults with T2D, acculturation is likely to be lower. Furthermore, compared with many other immigrant groups, Chinese retain their diet longer and more faithfully after immigration (44), with rice, noodles, ethnic breads, and starchy vegetables remaining staple foods in immigrant Chinese diets (45). One reason for this is that diet is at the center of life and tradition, with influences on emotions and interpersonal relations as well as health (46). At the nutrient level, compared with a western diet, a traditional Chinese diet contains large amounts of fiber and less saturated fats, being largely composed of vegetables, fruits, and meats (47) whereas a western diet contains more cholesterol and calcium (36).

Some of the cultural factors influencing nutritional treatment of T2D in Chinese immigrants to North America have been studied, mainly in the United States. In Chinese Americans with diabetes, family harmony and buy-in to the treatment regimen are critical to diabetes outcomes (48, 49). In the Chinese dietary tradition, the Yin-Yang (hot-cold) principles of food are deeply rooted, especially for older Chinese (34). A dependence on Chinese staple foods could be challenging to Chinese immigrants with T2D in western countries because these foods are not appreciated by western health service providers and educators (49). For example, a doctor’s recommendation to restrict rice consumption can lead to family conflict because rice brings a sense of well-being to Chinese people (49). Some Chinese Americans perceive that restriction of rice weakens their physical strength and keeps them from fulfilling their roles as a financial provider for the family (50). These findings suggest that recommended dietary changes for T2D have a huge influence on the emotional and physical health of Chinese Americans.

Lack of nutritional knowledge is also an issue for less-acculturated Chinese with T2D. Although most are aware of the importance of diet in managing their disease, they lack knowledge of portion sizes and food groups and continue to eat staple rice or wheat products daily (51). This leads to the consideration of health literacy of immigrant groups. Health literacy refers to “the wide range of skills and competencies that people develop over their lifetimes to seek out, comprehend, evaluate, and use health information and concepts to make informed choices, reduce health risks, and increase quality of life” (52). Among different aspects of health literacy, fundamental literacy and cultural literacy are of major concern in minority groups due to their language and cultural differences with the mainstream society.

English proficiency among immigrants highly influences their fundamental literacy, which includes speaking, reading, writing, and numeracy (52). Fundamental literacy is very important for diabetes management because there is a need to communicate with health professionals, read medication instructions, food labels, and other information about disease management. English proficiency is one of the most commonly cited indicators of acculturation. When immigrants come to North America, language is the first barrier to overcome (53). For those with a disease such as T2D, accessibility to medical services and disease management advice is limited by language capabilities, miscommunication, frustration, and distrust (50). It has been estimated that only half of Chinese Americans with T2D obtain advice on diabetes management (54), and those who speak little English are less likely to effectively monitor blood glucose (55). Lack of language capacity affects adherence to drug regimens and decreases knowledge of side-effects (51). Even when translation is provided, these services may not meet client needs (50, 56) and when family members serve as translators important medical or pharmaceutical information may not be fully understood because literal translation is not always appropriate in the delivery of medical information and health education (56). In order for effective health education and promotion, cultural competence is needed among health professionals and organizations. Culturally appropriate needs assessment is necessary before planning and implementing health education programs (57).

## General Acculturation Trends in Ethnic Chinese Populations in North America

Most Chinese immigrants in Canada reside in the largest metropolitan areas (58) and many reside in their own ethnic enclaves (59). For example, nearly half of the population of the Vancouver suburb of Richmond is ethnic Chinese (60). Studies of American Chinese indicate lower acculturation than for other immigrant groups (2) and less likelihood of speaking English (3). Approximately 15% of Chinese immigrants to Canada cannot speak either official language (English or French), although this is dependent upon age and education (58). Although the low acculturation is partially because of the collective orientation, individual differences among Chinese immigrants are significant in acculturation experience. Berry has suggested that individual socio-economic status and psychological factors affected acculturation experiences (61).

Variation in age, gender, and socio-economic status can make a difference in both acculturation experiences and outcomes. Korean immigrants who were younger and living in the US for 8 or more years were more acculturated than older people (62). Women were less-acculturated than men, which suggested a gender difference in acculturation levels (62, 63). Gender differences were attributed to men being more likely to work long hours and therefore more exposed to the American culture (62). Since the gender roles in family are similar in East Asian countries, this finding might be also valid in the Chinese American population and it was found that female Chinese Americans are less-acculturated than males (7). This gender-related difference in acculturation is reflected in health-related behaviors relevant to T2D, such as physical activity (64) because higher acculturation has been associated with higher physical activity (7). Women within households frequently place husbands’ and children’s preferences in front of their own when designing a meal; in contrast, men tend to emphasize their own creativity more than the taste preferences of those they serve (65). This gender difference may have influence on the nutritional aspects of diabetes management because diet is an important aspect of diabetes management. A female diabetes patient in a family might sacrifice meeting her own dietary requirements in order to satisfy the preferences of the husband and children. Thus, education or intervention programs may need to be targeted at the whole family or even larger environment to develop social support for individuals with diabetes.

With respect to education, Kandula and Lauderdale showed that Chinese Americans with education higher than high school were more likely to be acculturated and only 5.4% of Chinese having a high school education or less obtained acculturation score 3–5 (out of 5) using the SL-ASIA scale. They found a similar pattern of association between income and acculturation. People with a higher income were more likely to be highly acculturated than their counterparts with low income (64). These findings suggest that individual and socio-economic characteristics affected acculturation of Chinese immigrants in the United States, which may impact an individual’s ability to adopt a nutrition prescription for diabetes. The tendency for Chinese immigrants to live in segregated enclaves within the large metropolitan areas presents an opportunity to introduce culture-specific interventions within distinct geographical areas of a city to maximize reach and uptake of a program.

Although there is increasing research on the health practices of Chinese immigrants to North America, most focuses on traditional aspects of medical care of disease management. However, in Chinese culture, social and family influences are emphasized in disease management; an aspect little studied with regard to acculturation effects. Being influenced by the traditional collectivistic social orientation and the philosophy of harmony, Chinese Americans tend to value the well-being of the whole community and family more than individuals’ physiological and emotional health (48). In addition, changes in socio-economic status imposed by immigration may influence the community and family status of Chinese Americans. Therefore, these factors need to be considered in order to achieve optimal health care. For example, in traditional Chinese families filial piety and respect for elders is highly appreciated but are not valued in American culture; therefore, Chinese elders perceive less family support especially for diabetes management practices (50). Lower socio-economic status may lead to loss of face, depression, and changes in behavior such as reduced physical activity (50). Within the family, interpersonal harmony is an important value such that Chinese Americans may not express their negative emotions related to their disease in front of family members, who may in turn remain silent in order to avoid conflicts arising from altered family roles and responsibilities (49). Families or couples with higher interpersonal respect or conflict resolution skills have better disease management (66).

## Conclusions and Recommendations

There is an appreciation for the need to address cultural considerations when prescribing diets for the treatment of T2D to patients from ethnic minorities in North America in order to increase diet acceptability and adherence. Ethnic Chinese are a large minority population in North America. The degree of acculturation may impact dietary choices by ethnic Chinese but few studies have addressed this possibility directly, particularly in the segment of this population with T2D. In addition to diet, acculturation affects access to health care and the likelihood of engaging in other healthy behaviors such as physical activity. Furthermore, application of cultural considerations is complicated by the complexity of the acculturation process, which involves both personal and environmental factors. Social and family influences may be of particular importance in certain cultures.

The available literature does suggest a number of potential points of intervention that could improve dietary management of T2D in ethnic minorities in mixed societies such as Canada and the United States. Because immigrants tend to reside in cultural enclaves, targeted delivery of Chinese-language T2D programs in cities such as Vancouver or Toronto are both practical and a convenient testing ground for such enterprises. Indeed, health services listings for diabetes education in Toronto list the available languages at each clinic (www.diabetes.ca/files/Diabetes_Edc_cenTo.doc). Despite this, a study of immigrants to Toronto, including those speaking Mandarin, showed significantly lower glucose and foot checking behaviors compared with Canadian-born participants, although more immigrants did have regular physical activity and reduce carbohydrate intake. Immigrants were also less likely to access dietitians or other specialists (67). This may be due to overall low health literacy (52), which clearly must be addressed in order for optimal medical and behavioral treatment to occur. This outcome may be facilitated by increasing the cultural competency of health care providers. Assessment of the degree of dietary acculturation may also help but development and validation of instruments for use at the practice level would be required. For the population of immigrants not living in large metropolitan centers with readily available, multilingual health care services, increased availability of translated health education materials may be of benefit. However, one weakness of this approach is that texts are often translated literally. It is important to ensure that any adaptations of materials including both text and graphics have considered cultural differences and language nuances (52). Furthermore, it is necessary to go beyond simple translation of materials when considering nutrition programing. In order to increase diet acceptability, materials and program delivery should incorporate familiar foods that are available to participants, as well as appropriate cooking methods. The desirability of removing refined carbohydrates such as white rice from the diet of an ethnic Chinese patient with T2D may need to be tempered with the practicality of such a recommendation given the cultural connotations of such advice.

Finally, additional research is required in a number of focus areas. Gaps identified by Sanou et al. (1) are pertinent to the general nutritional health as well as to diets for diabetes and include assessment of risks and benefits of traditional foods as well as the practical aspects of how these can be included in a menu plan to maximize acceptability; strategies for maximizing access to health care services for immigrants; understanding the factors influencing food choice; and development, implementation, and evaluation of programs targeted at immigrant populations. In addition, cross-cultural evaluations are required so that an understanding of similarities and differences in acculturation processes between ethnic groups are appreciated.

## Conflict of Interest Statement

The authors declare that the research was conducted in the absence of any commercial or financial relationships that could be construed as a potential conflict of interest.
